# A voxel‐based morphometry study on gray matter correlates of need for cognition and exploratory information seeking

**DOI:** 10.1002/brb3.3138

**Published:** 2023-07-25

**Authors:** Serenella Tolomeo, Shermine Lau, Bindiya L. Ragunath, Peipei Setoh, Gianluca Esposito

**Affiliations:** ^1^ Institute of High Performance Computing Agency for Science, Technology and Research Singapore Singapore; ^2^ Psychology Program, School of Social Sciences Nanyang Technological University Singapore; ^3^ Department of Psychology and Cognitive Science University of Trento Rovereto Italy

**Keywords:** exploratory information seeking, information processing, intrinsic motivation, need for cognition, voxel‐based morphometry

## Abstract

**Background:**

Need for cognition (NFC) represents interindividual differences in tendencies to engage and enjoy cognitive endeavors. Exploratory information seeking (EIS) refers to individual tendencies to attain cognitive stimulation through acquiring information related to consumer products or services out of curiosity.

**Methods:**

The current study aims to provide an in‐depth investigation of the relationship between NFC and EIS and extend this relation to determine neuroanatomical correlates of NFC and EIS. This study proposed two central hypotheses: (1) NFC and EIS scores are positively correlated and (2) the gray matter volume (GMV) of brain regions implicated in motivation, valuation, and reward systems are positively associated with both NFC and EIS. Self‐report and structural MRI data of 91 Singaporean Chinese participants were utilized for the study.

**Results:**

No statistically significant correlation was revealed between NFC and EIS scores. Neuroanatomical associations of the GMV of brain regions implicated in visuospatial, attentional, and reward processing with individual constructs of interest were explored. When examining NFC and EIS scores, larger GMV in the right pallidum and left fusiform gyrus was found in participants that reported higher levels of NFC (vs. lower NFC levels), larger GMV in the left precuneus in those with greater tendencies to engage in EIS (vs. lower EIS levels), and larger GMV of the left fusiform gyrus associated with greater endorsement of both NFC and EIS. When investigating the exploratory factor analysis–generated factors of NFC and EIS, similar patterns of associations were found between self‐reported levels of agreement against factors and GMV of brain regions implicated.

**Conclusions:**

Correlational analysis and exploratory factor analysis indicated the absence of a relationship between NFC and EIS. Additionally, voxel‐based morphometry whole‐brain analysis revealed neuroanatomical correlates of the GMV of brain regions implicated in visuospatial, attentional, and reward processing with NFC and EIS.

## 1 INTRODUCTION

Historically conceptualized as a need to make sense of the world (Cohen et al., 1955), the original measure of need for cognition (NFC) closely interlinks an individual's inclination toward structure and clarity in the environment (Webster & Kruglanski, 1994). In 1982, NFC was reconceptualized to reflect a relatively stable inter‐individual difference (or personality trait) in general tendencies “to enjoy and engage in thinking” across a variety of areas and contexts (Cacioppo & Petty, 1982, p. 116; Cacioppo et al., 1996, 1984). Since then, NFC has been examined in a number of studies evaluating information processing (Cacioppo et al., 1996) personality (Fleischhauer et al., 2010; Hill et al., 2016) and cognitive variables (Fleischhauer et al., 2014; Mussel, 2013). As described in its conception, NFC can be compartmentalized into two central aspects in relation to thinking: (1) enjoyment––the stable intrinsic motive toward thinking; (2) engagement––the preference for tasks that demand effortful cognitive stimulation.

The “enjoyment” aspect of NFC highlights the expression of “need” in NFC, which signifies a reasonably stable intrinsic motivation in thinking (i.e., cognitive motivation) that is developed over time through individual experiences (Cacioppo & Petty, 1982; Cacioppo et al., 1996; Petty et al., 2009). Accordingly, the NFC accentuates individuals’ motivations to engage in cognitive processes (process‐focused) rather than making predictions of their cognitive outcome (i.e., ability, intelligence). According to Cacioppo and Petty (1982), individuals fall along a bipolar continuum of NFC, ranging from low to high cognitive motivation, in direct correspondence with their level of NFC. On the one hand, individuals low on NFC may consider thinking to be an unpleasant and laborious task that requires some form of incentive for them to engage in (i.e., low intrinsic or extrinsic motivation for thinking). On the other hand, individuals with a high NFC may consider the act of thinking as an activity that is enjoyable in and of itself and satisfies an underlying desire to think (i.e., high intrinsic motivation for thinking). Undeniably, research to date has provided ample empirical evidence in support of the enjoyment of the effortful thinking aspect of NFC.

In a similar yet distinct manner, another core aspect of NFC, “engagement” in thinking, pertains to the degree to which individuals allocate their mental resources to cognitive endeavors (i.e., cognitive effort). Compared to low‐NFC individuals, high‐NFC individuals more readily expend effort across an array of activities, not limited to information processing, logical reasoning, and problem‐solving (Cacioppo & Petty, 1982; Cacioppo et al., 1996).

Nevertheless, there seems to be a lack of agreement in the literature concerning the test dimensionality of NFC scales. While the widely used 34‐item NFC scale (Cacioppo & Petty, 1982) and its similarly valid shortened 18‐item version (Cacioppo et al., 1984) assume unidimensionality of NFC, the theoretical proposition of NFC and empirical evidence in the field indicates the presence of at least two core aspects measured by NFC. Consequently, the initial assertion of unidimensionality of the NFC scale by Cacioppo and Petty (1982) poses an issue to the representativeness of the scale in accurately reflecting underlying concepts of NFC. For instance, researchers that further investigated the dimensionality of NFC scales through factor analysis generated different numbers of subfactors inherent to NFC, including two factors (Forsterlee & Ho, 1999), three factors (Tanaka et al., 1988), and four factors (Lord et al., 2005). Thus, this raises the possibility of NFC having multiple dimensions. However, more recently, Hevey and colleagues (2012) tested the purported dimensions of NFC using confirmatory factor analysis and reported the best fit model for the one‐factor model with method effects associated with the valence of items (i.e., positive and negative wording). Thus, this provided support for the unidimensionality of NFC.

The exploratory buying behavior tendencies (Baumgartner & Steenkamp, 1996) refer to individual differences to engage in exploratory buying behavior. These tendencies are manifested in two distinguished facets: (1) exploratory acquisition of products (EAP); (2) exploratory information‐seeking (EIS), across different consumer contexts. EAP refers to individual tendencies to pursue sensory stimulation by means of risky and novel product preferences and varying purchases in consumption experiences. On the other hand, EIS refers to individual tendencies to attain cognitive stimulation by means of acquiring consumption‐related content, driven by curiosity.

Despite the lack of studies documenting the relationship between NFC and EIS, there is albeit just one study that provided evidence for the deterministic nature of NFC on EIS (Baumgartner & Steenkamp, 1996). The literature points to the general idea that NFC and EIS represent similar concepts at different levels of specificity, domain‐general and domain‐specific, respectively. Therefore, NFC may affect one's motivation to seek other avenues to satisfy their innate cognitive needs; seeking out information related to consumer products (i.e., EIS) is one of the many ways to fulfill one's NFC. Specifically, the interconnected, overlapping themes between NFC and EIS put forth the possibility that an individual's level of NFC might influence their level of endorsement of EIS.

At the rudimentary subconscious level, intrinsic motivation (and even curiosity) seems to be the underlying conceptual mechanism driving an individual's inclination to cognitive‐related processes in NFC as well as EIS (Baumgartner & Steenkamp, 1996; Cacioppo & Petty, 1982; Soubelet & Salthouse, 2017). Intrinsic motivation refers to an individual's inherent tendency to explore, learn, seek novelty and challenges, and extend and exercise their capacity (Ryan & Deci, 2000). Curiosity concerns an individual's desire to investigate, observe, and/or gather information, particularly when it involves something novel or interesting (Loewenstein, 1994).

In support of the psychometric properties of NFC, past literature reported that individuals high on NFC reported more *enjoyment* and a positive stance toward effortful endeavors (e.g., Cacioppo et al., 1996; Grass et al., 2017). Based on the conceptual definition, EIS is more specific to general information search about consumer products in fulfillment of cognitive stimulation needs. Individuals high on EIS have a natural, innate tendency to seek knowledge of consumption products out of curiosity (Baumgartner & Steenkamp, 1996). Moreover, EIS was found to be positively associated with the number of thoughts generated in response to an ambiguous and curiosity‐stimulating advertisement (Baumgartner & Steenkamp, 1996; Experiment 3).

At the behavioral level, NFC and EIS seem to predict information‐related behaviors. Past literature revealed individual differences in NFC concerning information‐related behavior (i.e., information‐processing, information‐seeking), where high NFC is generally associated with more effort utilized in search behavior extending to a wide variety of contexts and acquiring more related and elaborated information (Cacioppo et al., 1996). For instance, individuals with high NFC were more likely to actively seek more information and expend more cognitive effort to search for information beyond what was presented about a novel consumer product (Verplanken et al., 1992). Additionally, high‐NFC individuals tend to attain more knowledge from various sources, more critically evaluate the quality of information, and rely on all relevant information (Cacioppo et al., 1996). Moreover, Cacioppo et al. (1996) revealed that high‐NFC individuals tend to exhibit more information‐seeking across a variety of activities, issues (e.g., social issues), and current affairs (e.g., political debates). On top of that, Martin and colleagues (2005) found that individuals with high NFC (vs. low‐NFC) demonstrated greater preference for websites that contain more detailed information and use still images (rather than animations), suggesting preferential differences in types of information individuals with differing NFC look out for.

In comparison, the conception of EIS posits a general inclination to explore and learn more about the environment via information search and acquisition, with higher EIS scores associated with a greater extent of information sought (Baumgartner & Steenkamp, 1996; Experiment 4). While NFC concerns cognitive processing related to a wide array of activities (Cacioppo et al., 1996), EIS restricts information search to consumption‐relevant goods (Baumgartner & Steenkamp, 1996). Considering empirical evidence that suggests the implications of NFC in information‐related behaviors (e.g., Verplanken et al., 1992), it is likely that individuals with higher NFC would seek out more information. As such, EIS may be considered a behavioral manifestation of NFC in a context specific to consumerism, where the endorsement in aspects of information‐seeking (e.g., amount, type) is a function of NFC on EIS. Notably, intrinsic motivation and information‐seeking are concepts thought to be interwoven in the context of the current study (i.e., intrinsically motivated information‐seeking). While existing literature acknowledges that information‐seeking behaviors are complex and can be driven by different (and possibly a combination of) motives, such as curiosity, instrumental goals, positive emotional valence, and uncertainty reduction, the present study focuses on motives thought to play a role in intrinsic information‐seeking. Intrinsic information‐seeking reflects an individual's internal curiosity and inclination toward information in the absence of external motives or rewards (e.g., monetary gains) (Gottlieb et al., 2013). Accordingly, this perspective views the information‐seeking process to be in and of itself rewarding, typically driven by curiosity and non‐instrumental (i.e., without apparent outcome or goal) motives. Such a baffling nature of intrinsic information‐seeking raises the plausibility of the brain's involvement in such a process––where intrinsic rewards that are generated from specific regions of the brain assign values to information attained (Gottlieb et al., 2013).

Although past research documented extensive empirical research supporting NFC, they are primarily psychological in nature––there is a lack of neuroimaging studies directed at NFC‐specific processing. Moreover, there is a lack of empirical research revolving around EIS and the absence of brain imaging studies examining the underpinnings of EIS. Consequently, the neural basis of NFC and EIS are relatively undetermined and constructively speculated based on conceptual findings and assumptions of the specific variables.

Past neuroimaging studies on both humans and animals reported results that converge on the involvement of the orbitofrontal cortex (OFC), striatum, and other brain regions such as the amygdala and thalamus (Haber & Knutson, 2010), in extrinsic motivation and reward‐processing including primary, secondary, and social rewards (e.g., Smith & Delgado, 2015). The OFC plays a crucial role in evaluating the value and salience of different rewards, as well as in representing the expected outcomes of actions (Rudebeck et al., 2008). Lesion studies in animals have demonstrated that damage to the OFC can lead to impairments in reward‐guided behavior and decision‐making processes (Rolls, 2004). Furthermore, neuroimaging studies in humans have shown increased OFC activation during tasks involving reward anticipation and evaluation (Kringelbach & Rolls, 2004). The striatum forms a major subcomponent of the basal ganglia, which is located deep beneath the subcortical structures of the brain. It forms the main input stage of the basal ganglia, receiving information from all regions of the cortex, as well as from the limbic system, and increasing evidence in the field has suggested the role of the dorsal striatum in reward‐processing and decision‐making (e.g., Balleine et al., 2007). The amygdala, another important brain region, is involved in processing and regulating emotions, including those related to reward and motivation. It is known to play a crucial role in the formation and consolidation of emotional memories, as well as in the modulation of behavioral and physiological responses to rewarding stimuli (Davis & Whalen, 2001). Research has indicated that the amygdala receives inputs from various sensory systems and can integrate these inputs with information from other brain regions to generate appropriate emotional responses (LeDoux, 2000).

Despite the similar conceptual overlapping between NFC and EIS, only one existing study (i.e., Baumgartner & Steenkamp, 1996) directly examined their relationship. Furthermore, on top of the lack of and absence of neuroimaging‐related studies in NFC and EIS, respectively, it is to our notice that the neuroanatomical bases underlying individual differences in NFC and EIS, as well as their possible overlapping structures, have never been examined in past research. Thus, the goals of the current study are: (1) to picture the contrast between constructs of similar concepts such as NFC (domain‐general) and EIS (domain‐specific); (2) to strengthen the established relationship between NFC and EIS and extend this relation to establish overlapping structural brain regions related to both constructs using voxel‐based morphometry (VBM).

Based on previous psychological and neurobiological studies relevant to our constructs of interest (i.e., NFC and EIS), we tested the following hypotheses:

i. NFC and EIS scores are positively associated.

a. High NFC scores are associated with increased EIS scores.

b. Items measuring NFC and EIS converge on a similar subfactor. We further postulate that subfactors attained (if any) are positively associated with each other.

ii. Gray matter volume (GMV) brain structures implicated in motivation, valuation, and reward systems are positively associated with NFC and EIS.

The current study aims to investigate the relationship between NFC and EIS data and test hypotheses by conducting both behavioral (i.e., correlational tests and exploratory factor analysis) and neuroimaging (i.e., VBM whole‐brain analysis) analyses.

## 2 MATERIALS AND METHODS

### 2.1 Participants

A total of 82 participants between 21 and 41 years old were recruited for this study. Participants were either Singaporean Chinese students recruited from Nanyang Technological University (NTU) or Singaporean Chinese adults from the local community. Each participant was required to provide written informed consent before participating in the study in accordance with the Declaration of Helsinki. This method and procedure have been approved by the Institute Review Board of Nanyang Technological University (Protocol 2017‐01‐029).

Recruitment of participants was carried out in two phases differentiated by target sample––phase one concerns NTU students, while phase two engaged middle‐aged adults from the local community. Both phases employed identical inclusion and exclusion criteria. The inclusion criteria are as follows: (a) Chinese ethnicity, (b) English‐speaking, (c) right‐handed, (d) normal or corrected‐to‐normal vision and hearing, (e) no diagnosis of intellectual disabilities, (f) no psychiatric/neurological illness, and (g) no history of illicit drug use. Eligible participants were instructed to not consume any caffeine or medication 24 h prior to their scan. We excluded Chinese Singaporean participants who travelled overseas for more than 2 months over the past 6 months from the time of the scan session. In addition, with a particular focus on the safety of the participants, individuals with claustrophobia, any metallic prosthesis, and/or copper intrauterine devices were not eligible for the MRI section of the study. Participants were excluded if they did not fulfill the standard exclusion criteria for MRI. Finally, female participants at any stage of pregnancy were not eligible for the study.

### 2.2 Study design and procedure

#### 2.2.1 Need for cognition scale

The 34‐item Need for Cognition Scale (Cacioppo & Petty, 1982) was used to measure an individual's tendencies to engage in and enjoy thinking. Due to a technical glitch in survey collection software, responses to item 10, “The idea of relying on thought to make my way to the top does not appeal to me” were not recorded. Other example items include: “I really enjoy a task that involves coming up with new solutions to problems” and “I am usually tempted to put more thought into a task than the job minimally requires.” Items were rated on a 9‐point Likert scale (−4 = *very strong disagreement* to +4 = *very strong agreement*). Individual items were rescaled to represent a 5‐point Likert scale (1 = *strongly disagree* to 5 = *strongly agree*) to allow for better comparability between the two variables of interest. Specifically, individual items were rescaled in the following manner: −4, −3 = 1; −2, −1 = 2; 0 = 3; 1, 2 = 4; 3, 4 = 5. Negative items were also reverse coded. Scores were averaged to form a composite NFC score. Higher scores represent a higher NFC, while lower scores represent a lower NFC. The internal consistency reliability of NFC was strong (Cronbach α = .832).

#### 2.2.2 Exploratory information seeking subscale

The 10‐item exploratory information seeking (Baumgartner & Steenkamp, 1996) subscale adapted from the EBBT scale measures an individual's tendencies to attain cognitive stimulation through the acquisition of consumption‐relevant knowledge out of curiosity. Some examples of the items include: “I like to go window shopping and find out about the latest styles” and “I like to shop around and look at displays.” Items were scored on a 5‐point Likert scale (1 = *strongly disagree* to 5 = *strongly agree*). Reversed items were recoded, and scores were averaged to form a composite EIS score. Higher scores represent a higher EIS, whereas lower scores represent a lower EIS. The internal consistency reliability of NFC was strong (Cronbach α = .810).

### 2.3 Statistical analyses

#### 2.3.1 Statistical analyses of behavioral measures

All statistical analyses of behavioral measures (i.e., NFC and EIS) were executed on the Statistical Package for the Social Sciences (SPSS Version 26).

#### 2.3.2 Preliminary analysis

NFC and EIS data were first tested against the normality assumption with skewness and kurtosis. Results of normality assumptions were supplemented with the Shapiro–Wilk test. Possible gender effects were examined utilizing independent samples *t*‐tests.

#### 2.3.3 Partial correlation analysis

A partial correlation analysis was conducted to determine any existing relationships between NFC and EIS while controlling for age (and gender, should there be gender effects). Results with *p* < .05 were considered.

#### 2.3.4 Exploratory factor analysis

To further substantiate the relationship and understand the theoretical structure between NFC and EIS, an exploratory factor analysis (EFA) was conducted on the entire list of 43 items in NFC (33 items) and EIS (10 items). Before proceeding with factor analysis, several criteria should be met. First, the Kaiser–Meyer–Olkin measure of sampling adequacy (KMO) should exceed the Kaiser criterion of .50. Moreover, to pinpoint specific items that lacked multicollinearity, each item was assessed using the Measure of Sampling Adequacy (MSA). Only items that attained an MSA above .50 were retained. Last, Bartlett's test of sphericity should obtain a significant result at *p* < .05.

An EFA with principal axis factoring and Promax rotation was employed to determine the number of factors within the variables. All factors with an eigenvalue above 1 and the number of factors indicated before the scree plot graph flattens out were considered. To determine a factor structure that best fit the data, the practices identified by Costello and Osborne (2005) were adhered to in the analysis: (1) item loadings above ± .40; (2) no or few item cross‐loadings; (3) at least three items per factor. The factor structure with the best interpretability (i.e., “cleanest” factor structure) was retained.

The average inter‐item correlation, internal consistency reliability, and correlation of the factors were examined. Factors derived from EFA were also analyzed in association with VBM.

#### 2.3.5 Power analysis

Whole‐brain analyses were conducted on the brain regions to show associations with NFC, EIS, or both. As there has been a lack of published work measuring the neuroanatomical correlates of NFC and EIS in current literature, specific estimates about the effect size were assumed at a conservative level. As such, a medium effect size of *f* = 0.15 was specified for our analysis. To achieve a recommended power value of at least .80 at an alpha level of .05, a sample size of *N* = 55 was required. In addition, the test of correlation between NFC and EIS required a sample size of *N* = 67 to achieve at least a power of .80 at *p* < .05. Our study meets both criteria with a final sample size of *N* = 82.

#### 2.3.6 MRI

T1‐weighted high‐resolution anatomical images using magnetization‐prepared rapid acquisition gradient echo (MP‐RAGE) sequences (192 slices, TR = 2300 ms, TI = 900 ms, flip angle = 8°, and voxel size = 1 × 1 × 1 mm^3^) were collected from a 3‐Tesla whole‐body MRI scanner (Prisma; Siemens, Erlangen, Germany) with a 64‐element phased‐array head coil. All participants were fitted with a head restraint to prevent excessive head movement during the scan. No participant was found to have abnormalities in their brain structures.

#### 2.3.7 VBM pre‐processing

Structural MRI data were processed using Statistical Parametric Mapping 12 (SPM12; Wellcome Department of Imaging Neuroscience, http://www.fil.ion.ucl.ac.uk/spm/software/spm12) software package (Friston et al., 2007) (RRID: SCR_007037) in MATLAB 2020b platform (MathWorks, Natick, MA, USA). First, the T1‐weighted images were automatically segmented into different tissue types within the images (i.e., gray and white matter). The diffeomorphic anatomical registration through exponentiated lie algebra (DARTEL) technique was used to achieve more precision in the segmentation and normalization steps and inter‐subject registration into gray matter probability maps. Images were then spatially normalized with modulation to preserve the total amount of gray matter, then transformed into the Montreal Neurological Institute (MNI) stereotactic space to produce 1 × 1 × 1 mm^3^ voxels. Finally, they were smoothed by convolving the images with an isotropic Gaussian kernel of 8 mm full width at half maximum (FWHM; Ashburner & Friston, 2005).

#### 2.3.8 Behavior and neuroimaging correlations

In consideration of the ambiguous literature between NFC, EIS, and relevant neuroanatomical correlates, we investigated the association between GMV and scores in NFC and EIS using whole‐brain analysis with multiple regressions.

We intended to control total intracranial volume (TIV), age, and potentially gender as additional covariates in these analyses. TIV is of particular importance in volumetric structural neuroimaging measures as slight differences in a specific region of the brain may be confounded by individual differences in brain size (Mathalon et al., 1993; Whitwell et al., 2001). Besides, accounting for age was necessary for this study due to previously reported age differences in TIV (Bartholomeusz et al., 2002). More importantly, the analysis included participants from two different age groups, a young adult sample and a middle‐aged adult sample. Accordingly, differences in both self‐report and volumetric brains were expected between these two age groups; thus, including age in the regression model minimized confounds due to age differences. While Tanaka and colleagues (1988) detected gender differences in subfactor scores of NFC, the literature points to the fact that NFC is relatively gender‐neutral (Cacioppo et al., 1996). In response, preliminary independent *t*‐tests analyses were conducted to determine whether it is also necessary to control for possible gender effects. NFC and EIS scores were used as contrasts to test the significance of regressions coefficients from zero value.

Due to the lack of conclusive literature documenting associations between NFC and EIS on relevant brain structure volume, less stringent thresholds were used. Significance thresholds were set at a peak‐level threshold of *p* < .05 with family‐wise error (FWE) correction and uncorrected *p* < .0025 (as in Takeuchi et al., 2014) with an extended threshold of 20 contiguous voxels. Significant brain regions were anatomically defined and labeled in accordance with a brain atlas, automated anatomical labeling (AAL, Tzourio‐Mazoyer et al., 2002).

## 3 RESULTS

The descriptive statistics in the form of means, standard deviation, minimum, maximum, skewness, and kurtosis for demographic information (i.e., gender and age), as well as NFC and EIS scores for the final sample, are presented in Table 1. The kurtosis and skewness of individual items and overall scores on NFC and EIS scales fell within the absolute value of three (Table 1). Upon more stringent analysis, the Shapiro–Wilk test revealed a violation of the normality assumption for NFC scores, *W*(82) = 0.961, *p* = .014. Normality assumption of EIS scores was met, *W*(82) = 0.992, *p* = .879.

**TABLE 1:** Overall descriptive statistics across demographic information and variables of interest.

Variable	*n*	*M*	*SD*	Minimum	Maximum	Skewness	Kurtosis
Gender							
Female	43						
Male	39						
Age	82	25.8	5.539	20.0	42.0	1.463	0.895
NFC	82	3.296	0.446	2.091	4.394	0.490	0.521
EIS	82	3.096	0.706	1.3	5.0	0.119	0.190

Abbreviations: NFC, need for cognition; EIS, exploratory information seeking.

To examine possible effects of gender on NFC and EIS, two independent samples t‐test were conducted. No significant gender differences in NFC, *t*(80) = −0.008, *p* = .994, and EIS scores, *t*(80) = 1.829, *p* = .071. However, since EIS scores reached a marginal significance, we controlled for gender in the following analyses.

A partial correlation analysis was conducted to determine the relationship between an individual's NFC and EIS while controlling for age and gender (refer to Table 2). Results revealed no significant relationship between average NFC and EIS, *r* (78) = −.070, *n* = 82, *p* = .540.

**TABLE 2:** Partial correlation matrix between NFC and EIS.

Control	Variable	NFC	EIS
None	NFC	–	–
	EIS	–.054	–
Age and Gender	NFC	–	–
	EIS	–.070	–

Abbreviations: NFC, need for cognition; EIS, exploratory information seeking.

### 3.1 Factor analysis of NFC and EIS

**FIGURE 1:**
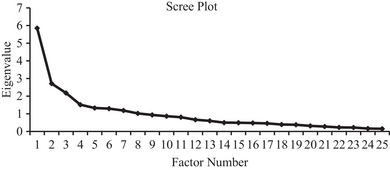
Scree plot for exploratory factor analysis with Promax rotation. *Note*: A total of 8 factors obtained an eigenvalue of above 1, before the graph flattens out.

We adopted a data‐driven approach (i.e., EFA) to better understand the underlying structure of the NFC and EIS. With principal axis factoring and Promax rotation, EFA was employed on the entire 43‐item list of NFC and EIS scales using SPSS version 26. The KMO generated was .508, meeting the Kaiser criterion of .50 needed for factor analysis (Kaiser, 1974). However, a total of 17 items (i.e., NFC: 3, 7, 8, 14, 19, 27, 28, 29, 31, 32, 33, 34; EIS: 11, 14, 16, 17, 19) attained an MSA below .50, indicating their lack of multicollinearity. After dropping the 17 items, the remaining items attained a KMO of .694, with all MSA above .50 except EIS item 13. With EIS item 13 excluded, the final list of items attained a KMO of .717, with all MSA well above .50. The Bartlett's test of sphericity revealed a significant result, *χ^2^* (300) = 762.528, *p* < .001, thus deeming it adequate to proceed with factor analysis. Factor analysis on the remaining 25 items revealed 8 factors with eigenvalues above 1 (Figure 1).

To determine the factor structure that best fits the data, a fixed number of pre‐specified factors (i.e., 1, 2, 3, 4, …, 7 factors) were extracted for further factor analyses. In accordance with practices laid down by Costello and Osborne (2005) (refer to EFA section 2.3.4 in Overview of Statistical Analysis), a three‐factor model solution that accounted for 42.949% of the total variance resulted in the “cleanest” factor structure (Table 3), with the most cohesive interpretation of factor content, was selected. Of note, the solution revealed a negative factor loading on item 11 of NFC and no factor loadings (i.e., small factor loadings below ± .40) on items 5, 12, 13, 15, and 23 of NFC. Thus, these items were removed for better factor interpretability. After these items were removed, the communality values obtained extended from .195 to .812, with a median value of .338. The factor loadings of individual items across each factor are presented in Table 4.

**TABLE 3:** Total variance explained by three‐factor model.

	Initial Eigenvalues			Extraction sums of squared loadings			Rotation sums of squared loadings
Factor	Total	% of Variance	Cumulative %	Total	% of Variance	Cumulative %	Total
1	5.848	23.392	23.392	5.228	20.911	20.911	4.828
2	2.713	10.850	34.242	2.300	9.200	30.111	3.471
3	2.177	8.706	42.949	1.604	6.416	36.526	2.346
4	1.517	6.068	49.016				
5	1.326	5.306	54.322				
6	1.293	5.173	59.495				
7	1.185	4.742	64.237				
8	1.018	4.071	68.308				
9	0.934	3.737	72.045				
10	0.864	3.455	75.500				
11	0.806	3.222	78.722				
12	0.665	2.661	81.383				
13	0.598	2.392	83.775				
14	0.503	2.010	85.785				
15	0.489	1.957	87.743				
16	0.481	1.925	89.667				
17	0.460	1.839	91.506				
18	0.395	1.581	93.087				
19	0.375	1.499	94.586				
20	0.311	1.244	95.831				
21	0.277	1.107	96.937				
22	0.229	0.917	97.854				
23	0.219	0.875	98.729				
24	0.167	0.667	99.396				
25	0.151	0.604	100.000				

**TABLE 4:** Pattern matrix of three‐factor model.

		Factor		
	Item	1	2	3
NFC_21	More often than not, more thinking just leads to more errors.*	**.831**	–.175	.002
NFC_22	I don't like to have the responsibility of handling a situation that requires a lot of thinking.*	**.713**	–.017	–.060
NFC_16	I prefer to think about small, daily projects to long‐term ones.*	**.629**	–.209	.042
NFC_26	I try to anticipate and avoid situations where there is a likely chance I will have to think in depth about something.*	**.596**	.142	.010
NFC_20	These days, I see little chance for performing mental effort.*	**.561**	–.076	.090
NFC_18	I find little satisfaction in deliberating hard and for long hours.*	**.543**	–.023	–.199
NFC_17	I would rather do something that requires little thought than something that is sure to challenge my thinking abilities.*	**.531**	.345	–.078
NFC_6	I am hesitant about making important decisions after thinking about them.*	**.464**	.042	.042
NFC_24	I feel relief rather than satisfaction after completing a task that required a lot of mental effort.*	**.457**	–.040	–.009
NFC_9	I have difficulty thinking in new and unfamiliar situations.*	**.445**	.097	.124
NFC_11	The notion of thinking abstractly is not appealing to me.*	–.427	–.082	–.145
NFC_5	Learning new ways to think doesn't excite me very much.*	.366	.261	–.087
NFC_15	I like tasks that require little thought once I've learned them.*	.351	.177	–.056
NFC_12	I am an intellectual.	.266	.164	–.013
NFC_1	I really enjoy a task that involves coming up with new solutions to problems.	–.200	**.881**	.024
NFC_2	I would prefer a task that is intellectual, difficult, and important to one that is somewhat important but does not require much thought.	–.034	**.615**	–.087
NFC_4	I am usually tempted to put more thought into a task than the job minimally requires.	–.102	**.569**	.011
NFC_30	I would prefer complex to simple problems.	–.032	**.522**	.145
NFC_25	Thinking is not my idea of fun.*	.162	**.485**	–.032
NFC_23	I appreciate opportunities to discover the strengths and weaknesses of my own reasoning.	.181	.272	.099
NFC_13	I only think as hard as I have to.*	.186	.270	–.003
EIS_18	I like to shop around and look at displays.	–.040	.001	**.897**
EIS_12	I like to go window shopping and find out about the latest styles.	–.011	.052	**.781**
EIS_15	I don't like to shop around just out of curiosity.*	.258	‐.019	**.704**
EIS_20	I often read advertisements just out of curiosity.	–.095	.038	**.513**

*Note*: Items are arranged in accordance to factor by factor loading size. Factor loadings of items retained are highlighted in bold. (*) denotes reversed items.

The items that converge on the same factor indicate that the three factors are specifically: (1) cognitive confidence (cogCon), (2) cognitive Complexity (cogCom), and (3) consumption‐related exploration (consumpExp). Cognitive confidence refers to an individuals’ level of confidence pertaining to engagement in cognitive activities. Cognitive complexity measures the extent to which individuals take preference and enjoy activities that command complex over simple processing. Consumption‐related exploration contains items that measure individuals’ tendencies to seek information related to consumption‐related products and/or activities. Labels and definitions for factors one and two were adapted from Tanaka et al. (1988) due to similarities in factor structures and content of NFC items within these subscales.

The average inter‐item correlations across factors were found to fall between .384 and .762, with an estimation of slightly lesser items across each factor falling out of the ideal range of .15 to .50 (Clark & Watson, 1995). Internal consistency reliability test revealed that all three factors achieved an acceptable reliability coefficient of > .60: cognitive confidence (10 items; α = .829), cognitive complexity (5 items; α = .727), and consumption‐related exploration (4 items; α = .802). The descriptive statistics in the form of means, standard deviation, minimum, maximum, skewness, and kurtosis across the three factors are presented in Table 5.

**TABLE 5:** Overall descriptive statistics for EFA‐generated factors.

Variable	*M*	*SD*	Minimum	Maximum	Skewness	Kurtosis
cogCon	3.088	0.730	1.40	5.00	0.144	−0.213
cogCom	3.612	0.730	1.80	5.00	−0.348	−0.108
consumpExp	3.262	0.925	1.00	5.00	−0.140	−0.041

Abbreviations: EFA, exploratory factor analysis, cogCon, cognitive confidence; cogCom, cognitive complexity; consumpExp, consumption‐related exploration; *M*, mean; *SD*, standard deviation.

Independent samples *t*‐test were conducted to examine possible effects of gender on the three factors. Gender difference was detected in consumption‐related exploration, with females (*M* = 3.459, *SD* = .873) scoring significantly higher than males (*M* = 3.045, *SD* = .942), *t*(80) = 2.068, *p* = .042. No significant gender differences were found in other factors (*p* > .05; refer to Table 6). As such, gender effects would be controlled for in the following analyses considering EFA‐generated factors.

**TABLE 6:** Independent samples *t‐*test illustrating gender effects across EFA‐generated factors.

	Female		Male				
Variable	*M*	*SD*	M	SD	*df*	*t*	*p*
cogCon	3.184	0.706	2.982	0.749	80	1.255	.213
cogCom	3.498	0.677	3.739	0.773	80	‐1.504	.137
consumpExp	3.459	0.873	3.045	0.942	80	2.068	.042

Abbreviations: EFA, exploratory factor analysis; cogCon, cognitive confidence; cogCom, cognitive complexity; consumpExp, consumption‐related exploration; *M*, mean; *SD*, standard deviation.

Correlation analysis revealed a significantly positive moderate correlation between cognitive confidence and cognitive complexity, *r*(80) = .333, *p* = .002. All other correlations were nonsignificant (*p* > .05; refer to Table 7). After controlling for age and gender, partial correlation revealed a similar pattern of associations between factors (refer to Table 7).

**TABLE 7:** Partial correlation matrix between factors generated by exploratory factor analysis.

Control	Factors	1	2	3
None	1. Cognitive confidence	–	–	–
	2. Cognitive complexity	.333**	–	–
	3. Consumption‐related exploration	–.029	–.008	–
Age and gender	1. Cognitive confidence	–	–	–
	2. Cognitive complexity	.364***	–	–
	3. Consumption‐related exploration	–.080	.027	–

* *p* ≤ .05; ** *p* ≤ .01; *** *p* ≤ .001.

The EFA revealed a factor structure in which items of NFC and EIS loaded on separate, uncorrelated factors, as opposed to what we expected. As such, results suggest a possibility that NFC and EIS might not share a similar underlying structure.

### 3.2 Whole‐brain analysis

**FIGURE 2:**
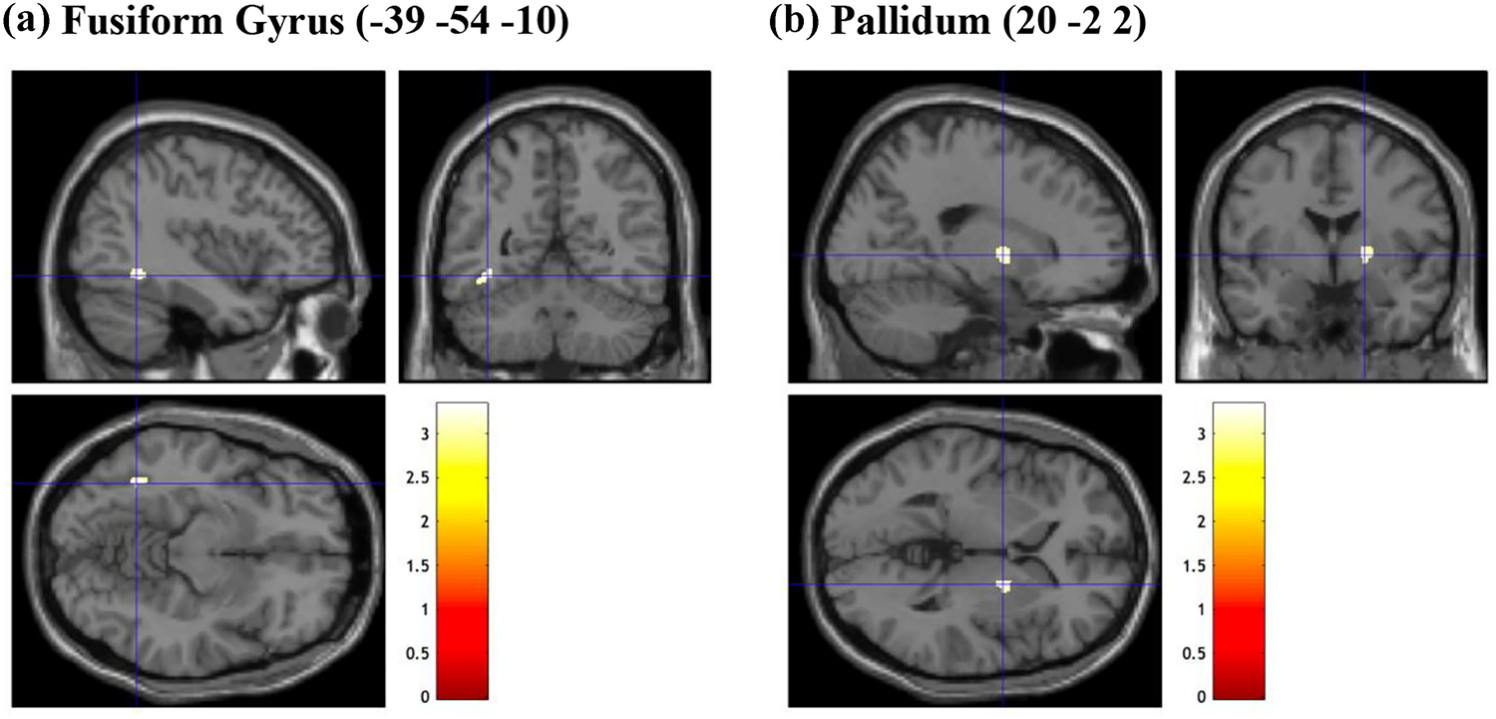
Brain sections illustrating GMV of regions related to NFC. (a) Fusiform gyrus (−39 −54 −10). (b) Pallidum (20 −2 2). NFC, need for cognition; GMV, gray matter volume. Intensity bar represents *T*‐value.

**FIGURE 3:**
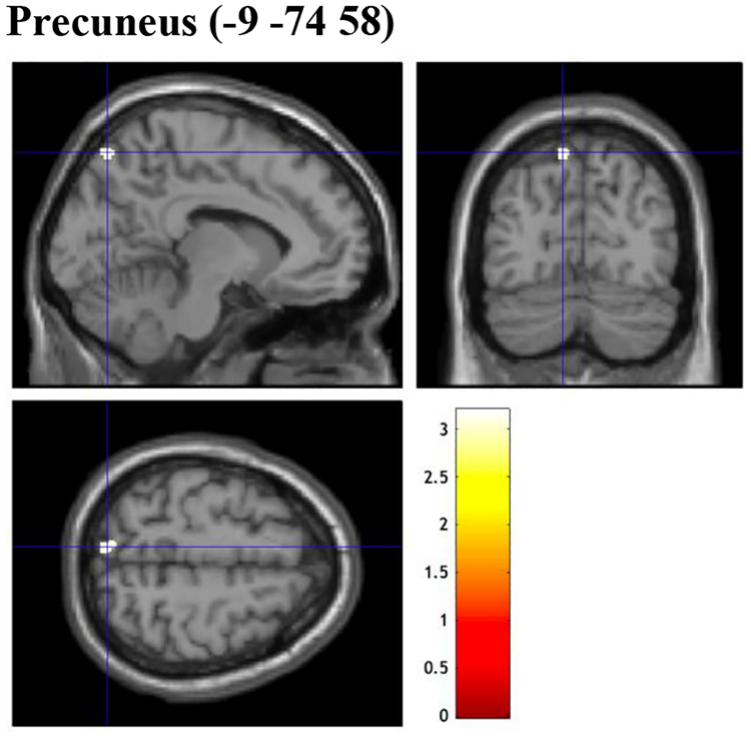
Brain sections illustrating GMV of regions related to EIS. Precuneus (−9 −74 58). EIS, exploratory information seeking; GMV, gray matter volume. Intensity bar represents *T*‐value.

**FIGURE 4:**
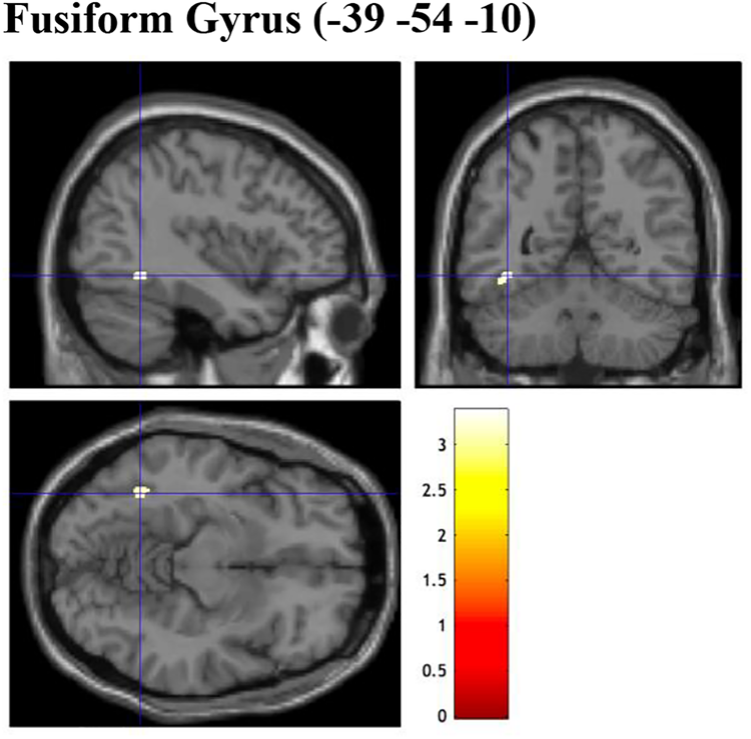
Brain sections illustrating GMV of regions related to both NFC and EIS. Fusiform Gyrus (−39 −54 −10). NFC, need for cognition; EIS, exploratory information seeking; GMV, gray matter volume. Intensity bar represents *T*‐value.

Several whole‐brain analyses were employed to examine the association between GMV and variables of interest, including NFC, EIS scales, as well as EFA‐generated factors. As the results of all whole‐brain analyses at a significant threshold of FWE‐correction at *p* < .05 revealed no suprathreshold clusters, the following section reports significant results with a significant threshold at uncorrected *p* < .0025 for multiple comparisons, with an extended threshold of 20 contiguous voxels. Whole‐brain analyses were conducted to investigate the association between GMV and scores in NFC (and EIS) scales, using multiple regression analyses. These analyses were performed considering TIV, age, and gender as additional covariates. The whole‐brain analysis revealed that NFC scores were positively associated with several regions of the brain, including the GMV of the left fusiform gyrus and right pallidum (uncorrected *p* = .001, Figure 2). A significant positive association was found between GMV of the left precuneus and the participant's EIS tendencies (uncorrected *p* = .001, Figure 3). When examining brain regions commonly associated with both NFC and EIS scores, our analysis revealed a positive association in GMV of the left fusiform gyrus (uncorrected *p* = .001, Figure 4). Table 8 details information on voxel size, peak MNI coordinates, *T*‐value, and anatomical location of implicated regions concerning NFC, EIS, and both.

**TABLE 8:** Location of brain area(s) related to NFC and EIS at peak‐level correction (uncorrected *p* < .0025, extent threshold = 20).

	Voxel size	MNI coordinates						
Contrast	(mm^3^)	x	y	z	*T* (76)	*p* (uncorr)	Side	Location (AAL)
NFC	340.875	−39	−54	−10	3.34	.001	L	Fusiform Gyrus
	367.875	20	−2	2	3.28	.001	R	Pallidum
	195.75	10	−20	−20	3.20	.001	–	–
EIS	178.875	−9	−74	58	3.19	.001	L	Precuneus
Common areas	239.625	−39	−54	−10	3.38	.001	L	Fusiform Gyrus

*Note*: Gray matter volume (GMV) of left fusiform gyrus and right pallidum were positively correlated to NFC, while GMV of left precuneus was positively correlated to EIS. GMV of the left fusiform gyrus was found to be positively associated with both NFC and EIS.

Abbreviations: NFC, need for cognition; EIS, exploratory information‐seeking; AAL, automated anatomical labeling.

**FIGURE 5:**
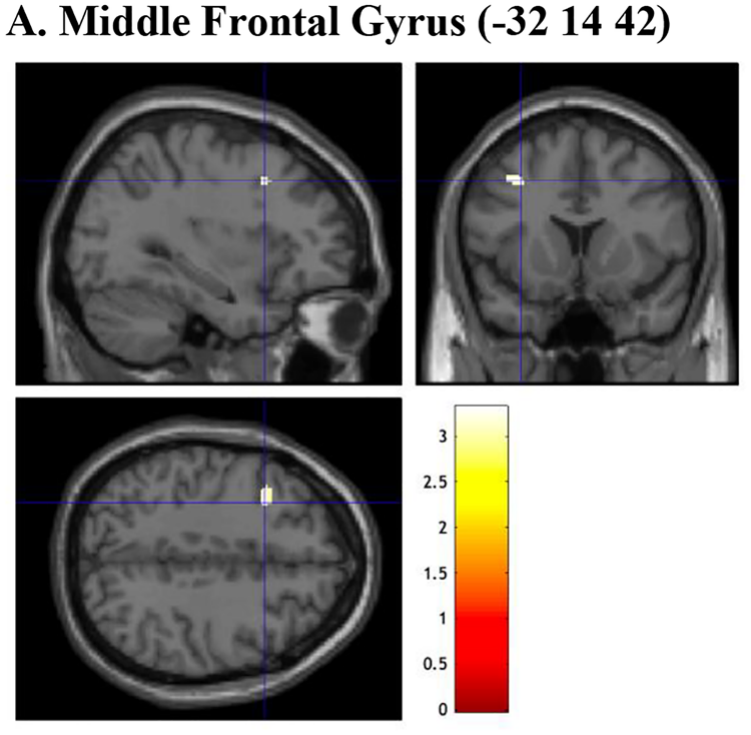
Brain sections illustrating GMV of regions related to cognitive confidence. (a) Middle frontal gyrus (−32 14 42). GMV, gray matter volume. Intensity bar represents *T*‐value.

**FIGURE 6:**
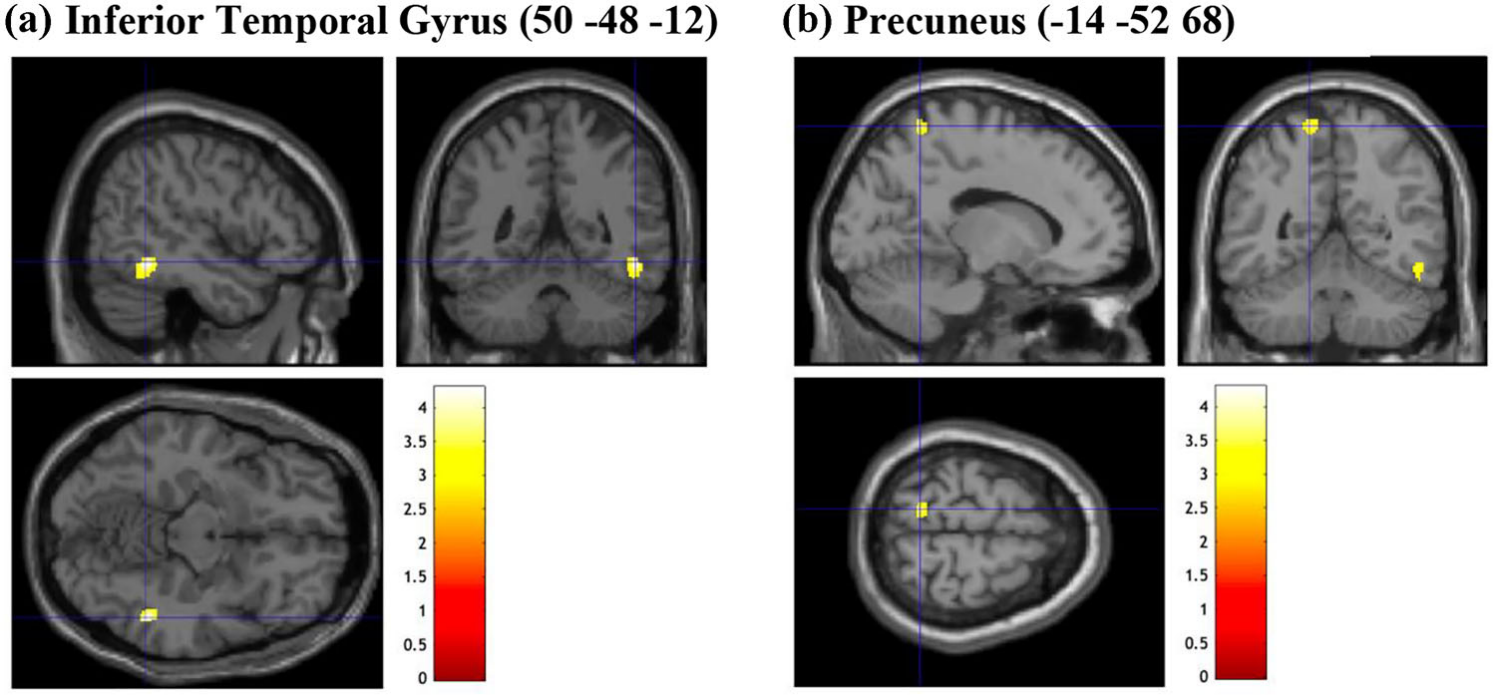
Brain sections illustrating GMV of regions related to cognitive complexity. (a) Inferior temporal gyrus (50 −48 −12). (b) Precuneus (−14 −52 68). GMV, gray matter volume. Intensity bar represents *T*‐value.

**FIGURE 7:**
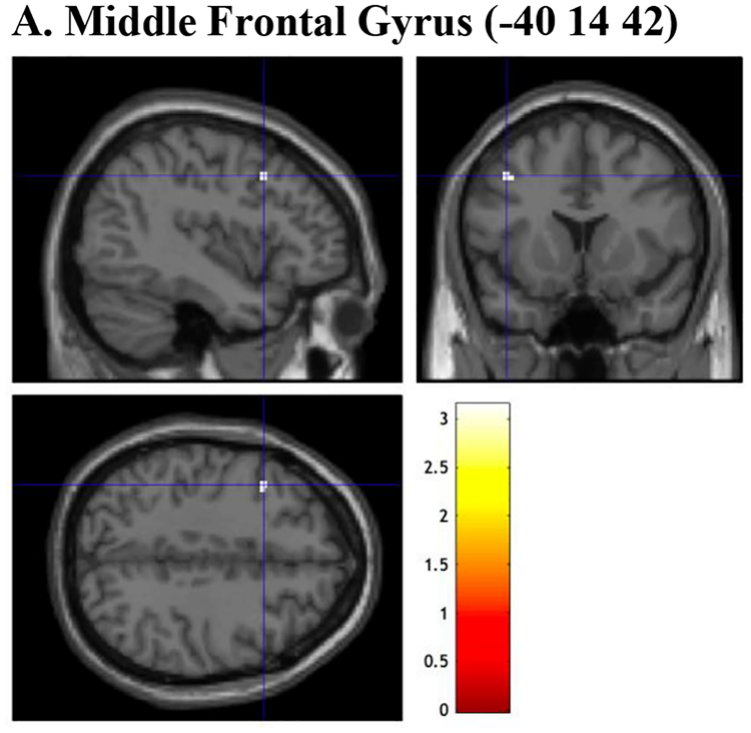
Brain sections illustrating GMV of regions related to consumption‐related exploration. (a) Middle frontal gyrus (−40 14 42). GMV, gray matter volume. Intensity bar represents *T*‐value.

**FIGURE 8:**
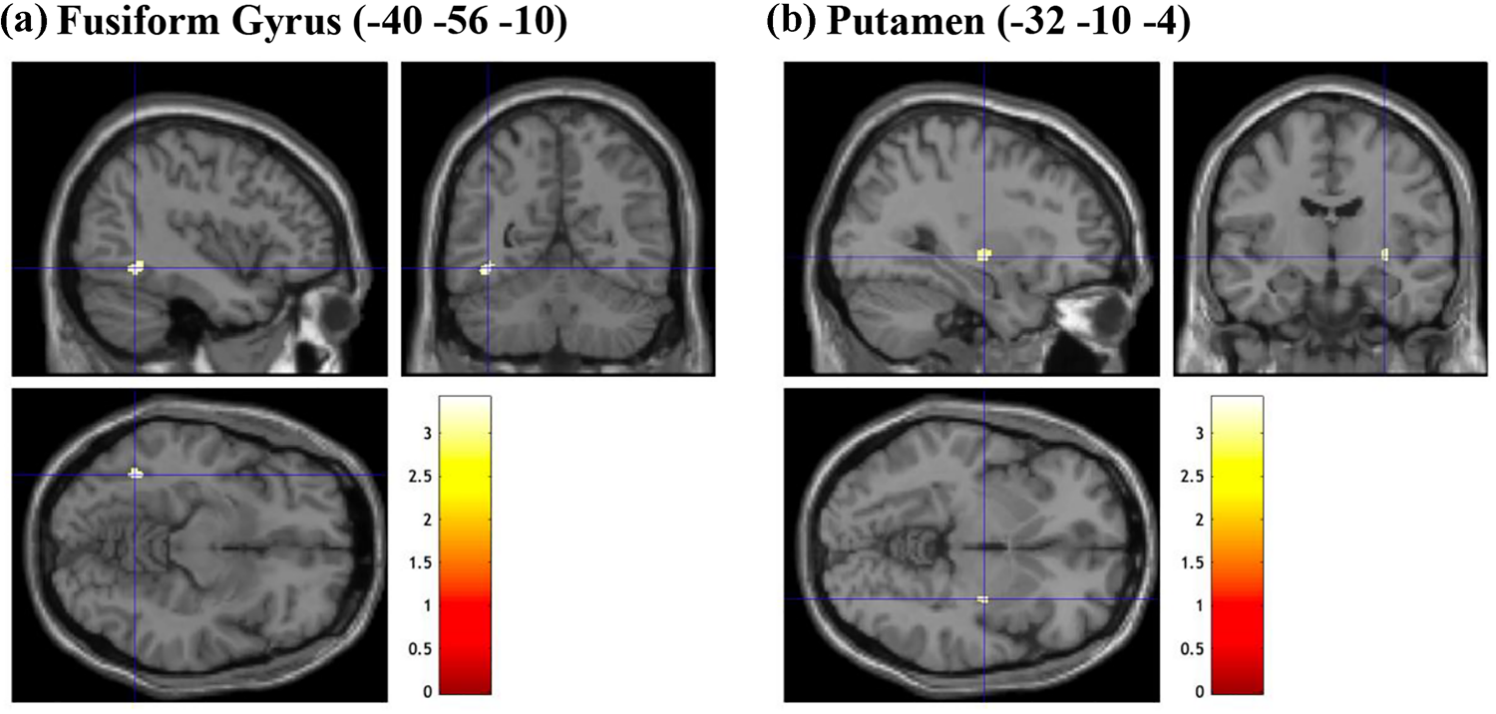
Brain sections illustrating GMV of regions related to all EFA‐generated factors. (a) Fusiform gyrus (−40 −56 −10). (b) Putamen (−32 −10 −4). EFA, exploratory factor analysis; GMV, gray matter volume. Intensity bar represents *T*‐value.

Group‐level whole‐brain analyses were also utilized to investigate the association between the GMV of the brain and the factors generated by the EFA (i.e., cognitive confidence, cognitive complexity, and consumption‐related exploration), using multiple regression analyses. Like the previous analysis, TIV, age, and gender were controlled for as covariates. Results revealed significant positive correlations between GMV of the left middle frontal gyrus and cognitive confidence (Figure 5), GMV of the right inferior temporal gyrus along with the left precuneus and cognitive complexity (Figure 6), and GMV of the left middle frontal gyrus and consumption‐related exploration (Figure 7), all uncorrected *p* ≤ .001. When the GMV jointly associated with all EFA‐generated factors was examined, significant positive correlations were found in the GMV of the left fusiform gyrus and the right putamen (Figure 8) (uncorrected *p* ≤ .001). Refer to Table 9 for detailed information on voxel size, peak MNI coordinates, *T*‐value, and anatomical location of implicated regions with respect to EFA‐generated factors.

**TABLE 9:** Location of brain area(s) related to factors generated by exploratory factor analysis (uncorrected *p* = .0025; extent threshold = 20).

	Voxel size	MNI coordinates						
Contrast	(mm^3^)	x	y	z	*T* (75)	*p* (uncorr)	Side	Location (AAL)
cogCon	256.500	−32	14	42	3.32	.001	L	Middle Frontal Gyrus
cogCom	823.500	50	−48	−12	4.29	<.001	R	Inferior Temporal Gyrus
	320.625	−14	−52	68	3.61	<.001	L	Precuneus
consumpExp	91.125	−40	14	42	3.15	.001	L	Middle Frontal Gyrus
Common Areas	232.875	−40	−56	−10	3.41	.001	L	Fusiform Gyrus
	155.250	32	−10	−4	3.08	.001	R	Putamen

*Note*: Positive associations were found between gray matter volume (GMV) of left middle frontal gyrus and cognitive confidence, GMV of right inferior temporal gyrus as well as left precuneus and cognitive complexity; GMV of left middle frontal gyrus and consumption‐related exploration; GMV of left fusiform gyrus as well as right putamen with cognitive confidence, cognitive complexity, and consumption‐related exploration.

Abbreviations: cogCon, cognitive confidence; cogCom, cognitive complexity; consumpExp, consumption‐related exploration; AAL, automated anatomical labeling.

## 4 DISCUSSION

The current paper investigated the underlying conceptual structures of NFC and EIS at two levels––behavioral and neuroanatomical. The main purpose of this study was to better understand the shared relationship and theoretical structure of conceptually similar constructs such as NFC (domain‐general) and EIS (domain‐specific), as well as to determine structural neuroanatomical correlates of both constructs previously neglected in the literature. We hypothesized a positive correlation between NFC and EIS, such that individuals with high NFC would have increased EIS. Given its conceptual overlaps, we further postulated that items measuring NFC and EIS would converge to form one coherent scale with positively correlated subfactors. We then extended our speculations to suggest the possibility of structural variations in individuals' GMV of brain regions related to motivation, reward‐processing, and value‐based processing in association with the magnitude of manifestations of NFC, EIS, and EFA‐generated factors. The findings provided partial support for our hypotheses.

### 4.1 Correlation and EFA between NFC and EIS

First, no statistically significant correlation was revealed between NFC and EIS scores, as opposed to the first assumption (H1a). When the underlying structures of the two variables were examined, the EFA revealed that NFC and EIS appear to be two independent factors, where items of NFC and EIS fall neatly into different subfactors. Of interest, the EFA further revealed two moderately correlated subfactors of NFC, opposing the unidimensional assertion of the NFC scale. Moreover, no statistical correlations were found between NFC and EIS items even at the subfactor level. These findings contradict existing literature that suggests a positive association between NFC and EIS (Baumgartner & Steenkamp, 1996) as well as the assumption that NFC and EIS held a common underlying basis.

One possible explanation for the absence of correlation could be that the participants generally reported neutral levels of NFC (*M* = 3.296; *SD* = .446) and EIS (*M* = 3.096; *SD* = .706). Accordingly, this is indicative of the clustering of scores, especially for NFC scores (Min. = 2.091, Max. = 4.394), around the neutral point, with very little spread of data.

Besides that, the lack of significant association could be explained by the rapid growth in consumer sectors, accompanied by technological advancements and the popularization of digital marketing. Such progressions ease the process of information search and information sharing, making it less demanding for individuals across populations, regardless of their differing NFC levels. In other words, EIS in the current century (as compared to 25 years ago) might not necessarily be considered an activity effortful enough to differentiate individuals with different levels of NFC but rather a leisure activity that individuals of all levels of NFC might enjoy. Moreover, with the increasing acceptance of digital marketing, people are more likely to turn to online shopping sites instead of traditional shops to satisfy their curiosity about consumption‐related information. As such, it is possible that the current EIS scale may not be the most fitting measure for curiosity‐induced consumption‐oriented information search in the current decade. These factors could potentially explain the lack of relationship between NFC and EIS, as well as the similar pattern of (the lack thereof) associations at the subfactor level.

### 4.2 Whole‐brain analysis

With respect to the whole‐brain analyses, the present study revealed neuroanatomical associations of the GMV of brain regions implicated in visuospatial, attentional, and reward processing with individual constructs of interest explored. When examining NFC and EIS scores, larger GMV in the right pallidum and left fusiform gyrus was found in participants that reported higher levels of NFC (vs. lower NFC levels), larger GMV in the left precuneus in those with greater tendencies to engage in EIS (vs. lower EIS levels), and larger GMV of the left fusiform gyrus associated with greater endorsement of both NFC and EIS. When investigating the EFA‐generated factors of NFC and EIS, similar patterns of associations were found between self‐reported levels of agreement against factors and GMV of brain regions implicated. Accordingly, larger GMV in the left middle frontal gyrus was found in participants that had more confidence to engage in cognitive activities, while larger GMV in the left precuneus was found in individuals that reported greater preference and enjoyment in complex cognitive processing (over simpler ones). Participants that reported higher levels of engagement in consumption‐related exploration had larger GMV in the left middle frontal gyrus. Last, larger GMV of the left fusiform gyrus and right putamen was found in individuals that reported greater endorsement across all EFA‐generated factors. Table 8 presents a side‐by‐side comparison of the GMV of brain regions that were positively associated with NFC and EIS against relevant subfactors generated by EFA. Interestingly, although GMV of brain regions positively associated with original and EFA‐generated NFC and EIS scores appears to be implicated in similar processes (i.e., visuospatial, attentional, and reward processing), structural variations of specific brain regions associated were relatively different. In the following paragraphs, anatomical and functional implications of related brain structures are explored and subsequently elaborated with relevance to the variables of interest in the current study.

The pallidum, composed of globus (dorsal) pallidus and ventral pallidum, comprises part of the lenticular nucleus located in the basal ganglia network. Owing to its anatomical placement and shared functional pathway with the ventral striatum, both nucleus accumbens and ventral pallidum were understood to receive inputs connecting limbic structures (Sesack & Grace, 2010). A growing body of research has gathered evidence to suggest the vital function of the (previously neglected structure) ventral pallidum in reward‐related processing (Smith et al., 2009), asserting its role as a reward‐processing center. It is to no alarm that larger GMV of the pallidum was associated with individuals thought to have greater inclinations to enjoy and react positively to cognitively demanding activities (i.e., higher NFC).

Located in the inferior temporal cortex in the brain, the fusiform gyrus sits at the basal surface of the temporal and occipital lobes and between the parahippocampal gyrus and medially from the lingual gyrus (Palejwala et al., 2020). While precise functions of the fusiform gyrus remain contested, research revealed its implications in the recognition and higher‐order processing of visual information. This includes recognition and/or processing of color (e.g., Simmons et al., 2007), face (e.g., Weiner & Grill‐Spector., 2012; fusiform face area), body (e.g., Peelen & Downing, 2005), language, words, and within‐category identification of stimuli (e.g., McCandliss et al., 2003; visual word form area), as well as high spatial frequency stimuli (e.g., Roberts et al., 2013). As such, the fusiform gyrus, in the current context, may take on a more fundamental role, functioning as an underlying brain structure that recognizes and processes different components of visual information in the environment, integrating information of all forms.

The precuneus, positioned at the posterior region of the parietal cortex, is thought to perform a wide range of integrated processes, including visuospatial processing (e.g., ability to perceive, process, and manipulate visual patterns and imageries), retrieval of episodic memory, and sense of self and agency (Cavanna & Trimble, 2006). Of relevance to visuospatial imagery processing, the precuneus has been implicated in attentional processes, typically showing greater activations in tasks involving direct attention (e.g., Simon et al., 2002) and attentional shifts (Le et al., 1998; Nagahama et al., 1999). For its contribution to attentional processes, the more elaborated precuneus volume in participants with higher EIS tendencies as well as in those with greater preference for complex activities may be primarily implicated to allow for better precision in the general processing of information.

Positioned between the inferior and superior frontal gyrus, the middle frontal gyrus forms the largest structure among the three frontal gyri. Past literature has demonstrated the imperative role of the middle frontal gyrus in attentional reorientation (Japee et al., 2015), working memory (Klingberg et al., 1997), as well as in the comprehension of speech and language (Hazem et al., 2021). Notably, the middle frontal gyrus, particularly in the right hemisphere, has been asserted for its fundamental role as a center for regulating attentional processes (e.g., Japee et al., 2015; Thiel et al., 2004), acting as the key hub between ventral and dorsal attention network (Vossel et al., 2014). Broadly speaking, the ventral network has been implicated in bottom‐up visual attentional processing (e.g., detection of unexpected stimuli, attentional shifts), while the dorsal network has been linked to governing top‐down visual attentional processing (e.g., deliberate allocation of attention to specific stimuli) (Vossel et al., 2014). Besides that, activation in the left middle frontal gyrus had also been detected in association with reorienting of attention (e.g., Thiel et al., 2004), putting forth the potential of bilateral involvement of the middle frontal gyrus in attentional processes. As with the precuneus, the middle frontal gyrus, in the current setting, may be involved in more efficient focusing of attention to information of interest in the environment.

The inferior temporal gyrus is one of three gyri located most ventrally on the lateral surface of the temporal lobe. Mounting research has identified the role of the inferior temporal gyrus in higher‐order cognitive operations (see Lin et al., 2020), suggesting its contributions to semantic memory (Smith & Squire, 2009), language (Acheson & Hagoort, 2013), visual perception (Buckley et al., 1997; Herath et al., 2001; Ishai et al., 1999), and multisensory integration (Mesulam, 1998). Of particular relevance, patients with chronic schizophrenia were found to have reduced bilateral volume in the inferior temporal gyrus (Onitsuka et al., 2004), implying that deficits in the inferior temporal gyrus could potentially result in impairment in multisensory semantic integration and complex visual perception (Tek et al., 2002). Putting into context, the functionality of the inferior temporal gyrus seems to be of great relevance to the behavioral expression of cognitive complexity, defined in the study as an individual's tendency to prefer and enjoy complex over simple processing, particularly suggesting the role of the inferior temporal gyrus in its facilitation of more cognitive demanding activities, and the further integration of gathered information, before encoding the value of information.

Situated in the basal ganglia, the putamen (part of the dorsal striatum) has been recognized for their direct involvement in reward and decision‐making (Belleine et al., 2007), with particular implications in action‐contingent learning. While past studies on humans (and animals) highlighted the distinct role of the dorsal striatum specific to learning about actions and their corresponding rewards (e.g., O'Doherty, 2004; Yin et al., 2005), the putamen and caudate have been suggested to take on separate roles in such process. Respectively, the putamen and caudate nucleus have been implicated in the coding of stimulus‐action association and reward‐prediction errors (i.e., the disparity between predicted and obtained reward) (e.g., Haruno & Kawato, 2006). Consequently, the enlarged putamen volume jointly associated with higher scores across EFA‐generated factors may play a role in assigning rewards to past enjoyable experiences relevant to engaging in demanding activities (in NFC) or searching for information related to consumer products (in EIS).

As a matter of fact, humans typically gain input of information via visual and/or auditory means. Though the role of brain regions associated with visual processing was not explicitly considered in the current study, it is not surprising that individuals with higher NFC and EIS tendencies are “better equipped” in their visual processing regions. Based on previous literature, it is likely that individuals with a greater inclination towards information‐seeking (indirectly implied by higher levels of NFC and EIS, especially NFC) would demonstrate greater alertness to their environment, accompanied by greater processing of finer visual information, allowing for an increased propensity to gain more useful information. The underlying motive, the process of gathering information, as well as the process of encoding values of information, are thought to be rewarding, thus, resulting in implications with rewards structures.

One limitation of the current study concerns the cross‐sectional nature of the study design, which poses restrictions to any implication of causality. For instance, while a positive correlation between GMV of right pallidum and NFC score was revealed in the whole‐brain analysis, the directionality of effects cannot be implied. In other words, there is no definitive method (in the context of the current study) that we can employ to conclude whether it is the larger GMV in the pallidum that causes higher NFC tendencies or specific behaviors and processing engaged in high NFC individuals that resulted in enlarged GMV in the pallidum. Notably, these GMV results do not have a direct relationship with functional activity or processing capacity. Therefore, future research could utilize longitudinal studies to illustrate trends and changes in structural brain volume in association with variables of interest (e.g., NFC) across time as well as functional activity changes. This allowed for more indicative results of possible causal effects regarding the variables of interest on the specific brain region.

Nevertheless, it is to note that information behaviors (e.g., amount of information needed, used, and sought), though represented a more direct outcome to both NFC and EIS and concern to the theoretical formulation of the current study, were not directly examined presently. As a consequence, information‐seeking inclinations could only be assumed by higher reported levels of NFC and EIS. Furthermore, it is important to acknowledge that the factor labels used in this study (NFC and EIS) were employed to provide a conceptual framework for interpreting the results and relating them to the reader. While these labels were based on similarities with prior literature, it is crucial to recognize that they represent broad constructs that encompass various cognitive and behavioral aspects. The factors themselves were derived from the factor analysis, and the association with specific information behaviors was assumed based on higher reported levels of NFC and EIS. However, it is important to note that the specific relationship between the factor labels and information behaviors was not directly examined in this study. Future research should consider incorporating measures of information behaviors to provide a more nuanced understanding of how these factors relate to specific information‐seeking tendencies and behaviors.

Lastly, the results interpreted in the current study were based on a homogeneous sample of Asian Chinese participants, consequently placing limits on the generalizability of the finding to other populations (e.g., Caucasians). Thus, future research could use a more diverse sample to understand NFC and EIS in other contexts.

## 5 CONCLUSION

To the best of my knowledge, this is the first study that conducted an in‐depth investigation of the associations between NFC and EIS and how they may contribute to GMV and constructs relevant to intrinsically motivated information‐seeking (i.e., NFC, EIS, and EFA‐generated factors). The current study revealed distinct and shared regions underlying similar processes associated with NFC and EIS, providing evidence for the presence of reward‐processing in NFC and EIS, and highlighting the importance of visuospatial and attentional processing systems in association with NFC and EIS behaviors.

## CONFLICT OF INTEREST STATEMENT

The authors declare that they have no known competing financial interests or personal relationships that could have appeared to influence the work reported in this paper.

### PEER REVIEW

The peer review history for this article is available at https://publons.com/publon/10.1002/brb3.3138.

## Data Availability

The data that support the findings of this study are available from the corresponding author upon request.
